# Supplementary material for: Impact of Yeast-Derived β-Glucans on the Porcine Gut Microbiota and Immune System in Early Life
Source: Microorganisms. 2020 Oct 13;8(10):1573. doi: 10.3390/microorganisms8101573 (PMC7601942; doi:10.3390/microorganisms8101573)
Supplement: Supplementary file 1 [file microorganisms-08-01573-s001.zip › 20200831 - Supplementary figures including captions.docx]

Supplementary Materials

**Table S1.** Experimental diets fed during the experimental period.

|  | **Weaner Diet**  Day 25-44 | **Nursery Diet**  Day 45-70 |
| --- | --- | --- |
| *Ingredient composition (%)* |  |  |
| Barley | 25.00 | 30.00 |
| Wheat | 25.00 | 22.00 |
| Corn | 15.28 | 12.00 |
| Soybean meal (48% crude protein) | 10.00 | 9.90 |
| Sweet whey powder | 9.29 | 4.29 |
| Soy protein concentrate ^1^ | 4.30 | 4.00 |
| Soya oil | 3.20 | 3.31 |
| Cane molasses (> 47.5% sugar) | 1.00 | 1.00 |
| Sucrose | 1.50 | 1.50 |
| Wheat bran | 1.50 | 5.13 |
| Sunflower seed meal (27% crude protein) | 0 | 3.00 |
| Sodium chloride | 0.58 | 0.46 |
| Sodium bicarbonate | 0 | 0.29 |
| Mono-calcium phosphate | 0.29 | 0.03 |
| Limestone (calcium carbonate) | 0.47 | 0.56 |
| Organic acids ^2^ | 0.15 | 0.15 |
| Phytase ^3^ | 0.05 | 0.05 |
| Vitamins and trace minerals ^4^ | 1.05 | 1.12 |
| Synthetic amino acids | 1.34 | 1.21 |
| Total | 100.00 | 100.00 |
|  |  |  |
| *Calculated nutrients, g/kg* |  |  |
| Moisture | 110 | 113 |
| Crude protein | 170 | 175 |
| Crude fat | 50 | 52 |
| Crude fibre | 28 | 40 |
| Crude ash | 47 | 47 |
| Starch (Ewers method) | 384 | 378 |
| Total dietary fibre | 141 | 165 |
| Soluble dietary fibre | 64 | 79 |
| Insoluble dietary fibre | 80 | 94 |
| Lactose | 65 | 30 |
| Calcium | 5.50 | 5.50 |
| Phosphorus | 4.50 | 4.36 |
| Digestible Phosphorus | 4.10 | 3.50 |
| Na | 3.00 | 3.00 |
| Cu (total, mg) | 165 | 167 |
| Zn (total, mg) | 124 | 129 |
| Metabolic energy (MJ) | 14.22 | 13.84 |
| Net energy (MJ) | 10.46 | 10.11 |
| Standardized ileal digestible lysine | 12.35 | 12.03 |
| *Analysed nutrients, g/kg* |  |  |
| Moisture | 101 | 97 |
| Crude protein | 170 | 176 |
| Crude fibre | 28 | 40 |
| Crude fat | 53 | 53 |
| Crude ash | 46 | 47 |
| Zinc (mg/kg) | 118 | 116 |

^1^HP 300 (Hamlet protein, Horsens, Denmark); ^2^ Fylax Forte HC-SP (Trouw Nutrition Selko, Tilburg, The Netherlands) ^3^ Phyzyme XP 5000 TPT (Danisco Animal Nutrition, Marlbourough, UK ) providing 600 FTU 6-phytase per kg feed; ^4^ Farmix (Trouw Nutrition, Putten, The Netherlands), provided per kg feed: 8000 IU vit A, 2000 IU vit D3, 100 (weaner) or 150 (nursery) IU vit E-acetate, 1.5 mg menadione, 1 mg thiamine mononitrate, 4 mg riboflavin, 1 mg pyridoxine, 30 µg cyanocobalamin, 20 mg niacin, 12 mg pantothenic acid, 300 µg folic acid, 150 mg choline chloride, 50 mg betain.


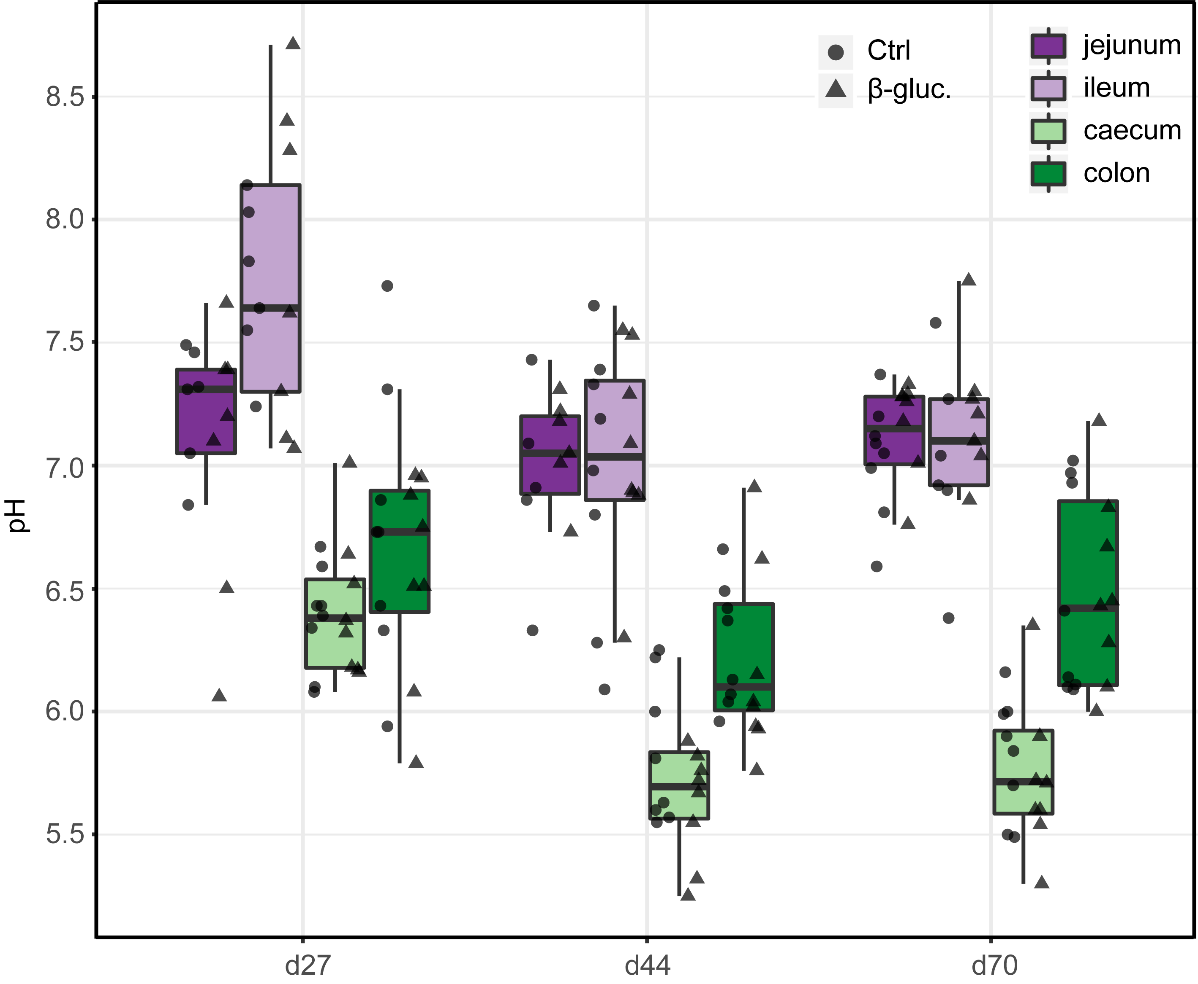


**Figure S1.** The pH of digesta from four different gut segments (jejunum, ileum, caecum and colon). The pH of digesta was measured on day 27, 44 and 70 of the study. Samples from control animals (circles; ●) and β-glucan treated animals (triangles; ▲) are presented in this figure (n = 8 per group).


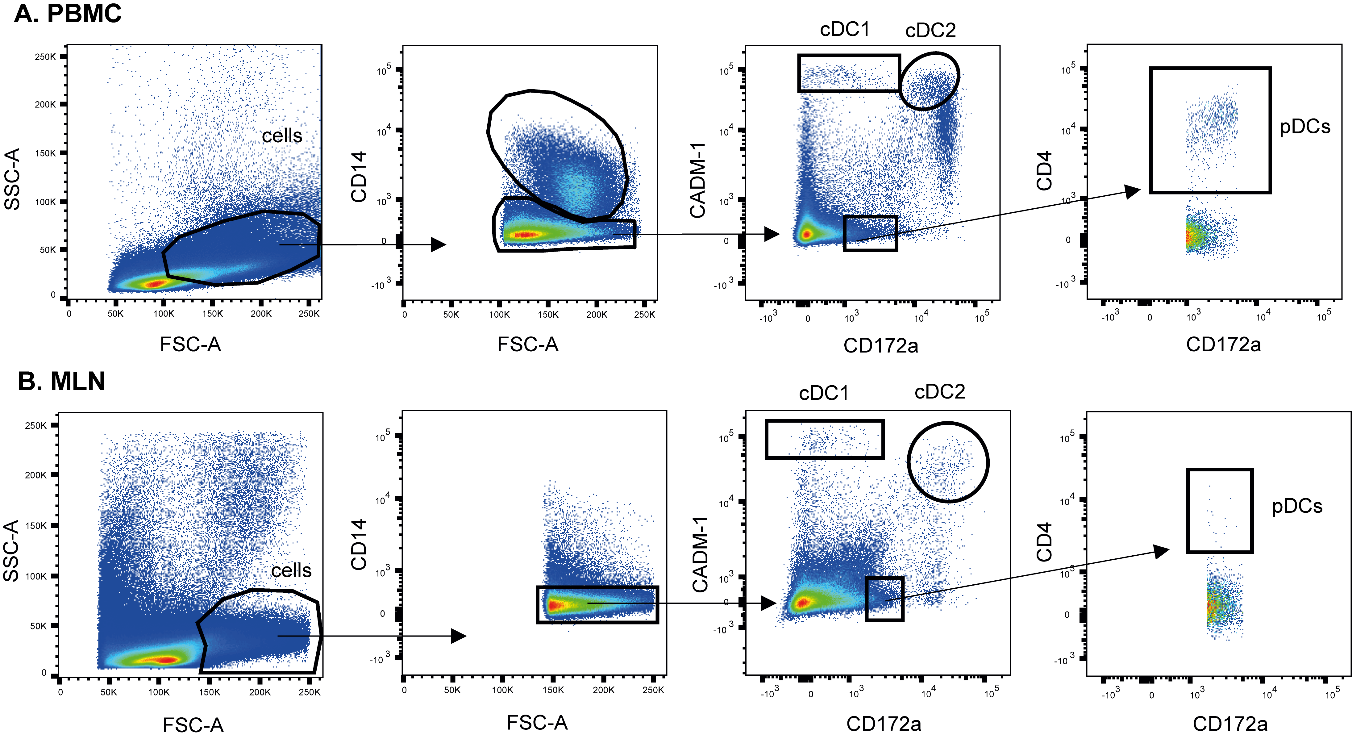


**Figure S2.** Gating strategy for the identification of DC subsets following five-color flow cytometry. Antibodies against CD14, CD172a, CADM1 and CD4 in were used to identify DC subsets in PBMCs (A) and MLN cells (B). After doublet discrimination and selection for viable cells, gates were set on cells with high forward and side scatter (large cells). DC subsets (CD14−), were defined as pDC (CD172a+CADM1-CD4+), cDC1 (CD172alowCADM1+CD4− cells) and cDC2 (CD172a+CADM1+CD4−).


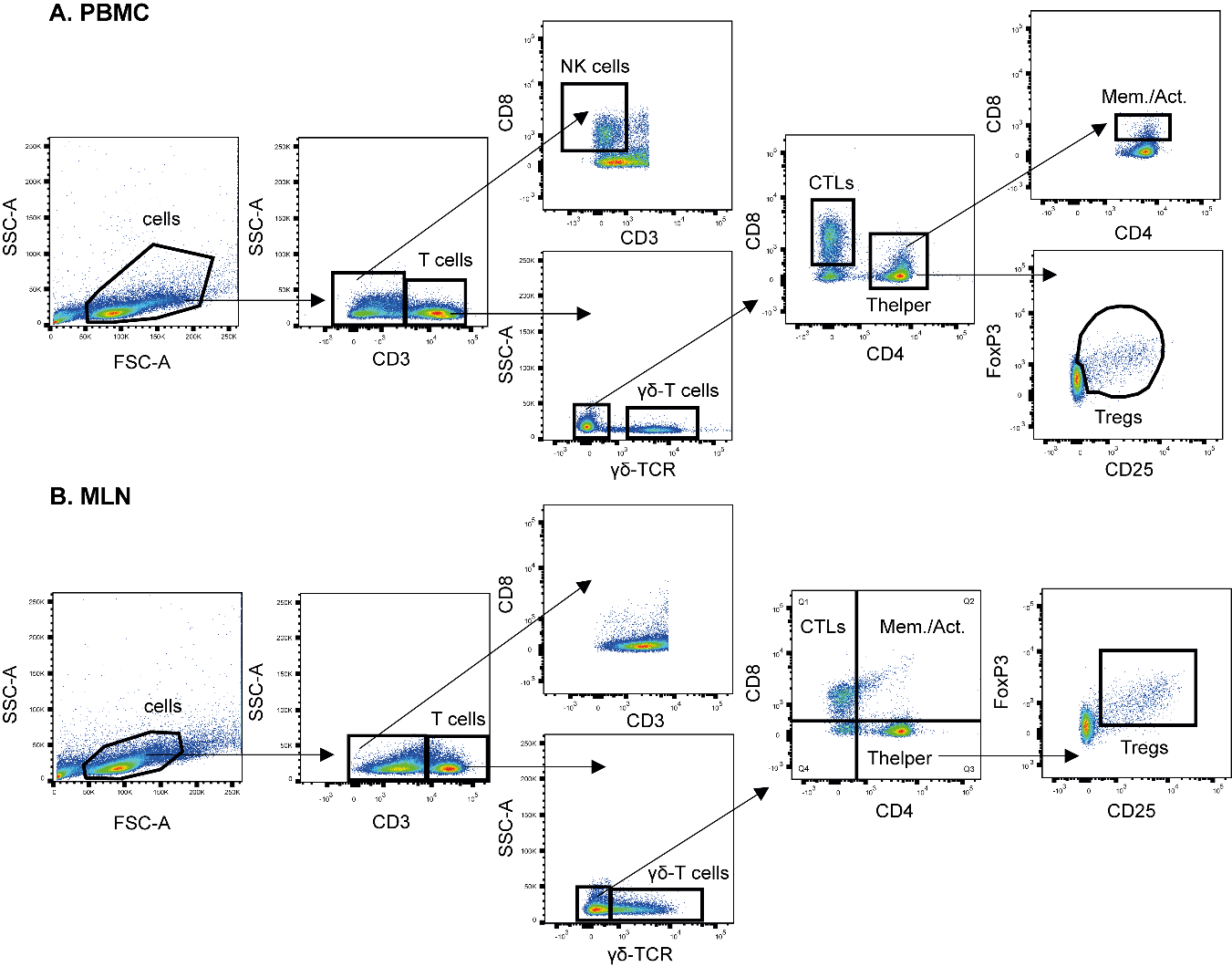


**Figure S3.** Gating strategy for the identification of T lymphocytes and NK cells following six-color flow cytometry. Antibodies against CD3, CD8α, TCR-γδ, CD4, FoxP3 and CD25 were used to identify different cell populations in PBMCs (A) and MLN cells (B). After doublet discrimination and selection for viable cells, gates were set on cells with medium/high forward and side scatter to select for lymphocytes and exclude debris. NK cells were defined as CD3-CD8α+, γδ T cells as CD3+TCR-γδ+, CTLs as CD3+TCR-γδ-CD8a+, T helper cells as CD3+TCR-γδ-CD4+, Memory/Activated (Mem./Act.) T cells as CD3+TCR-γδ-CD4+CD8a+ and T regulatory cells (Tregs) as CD3+TCR-γδ-CD4+CD25highFoxp3+.


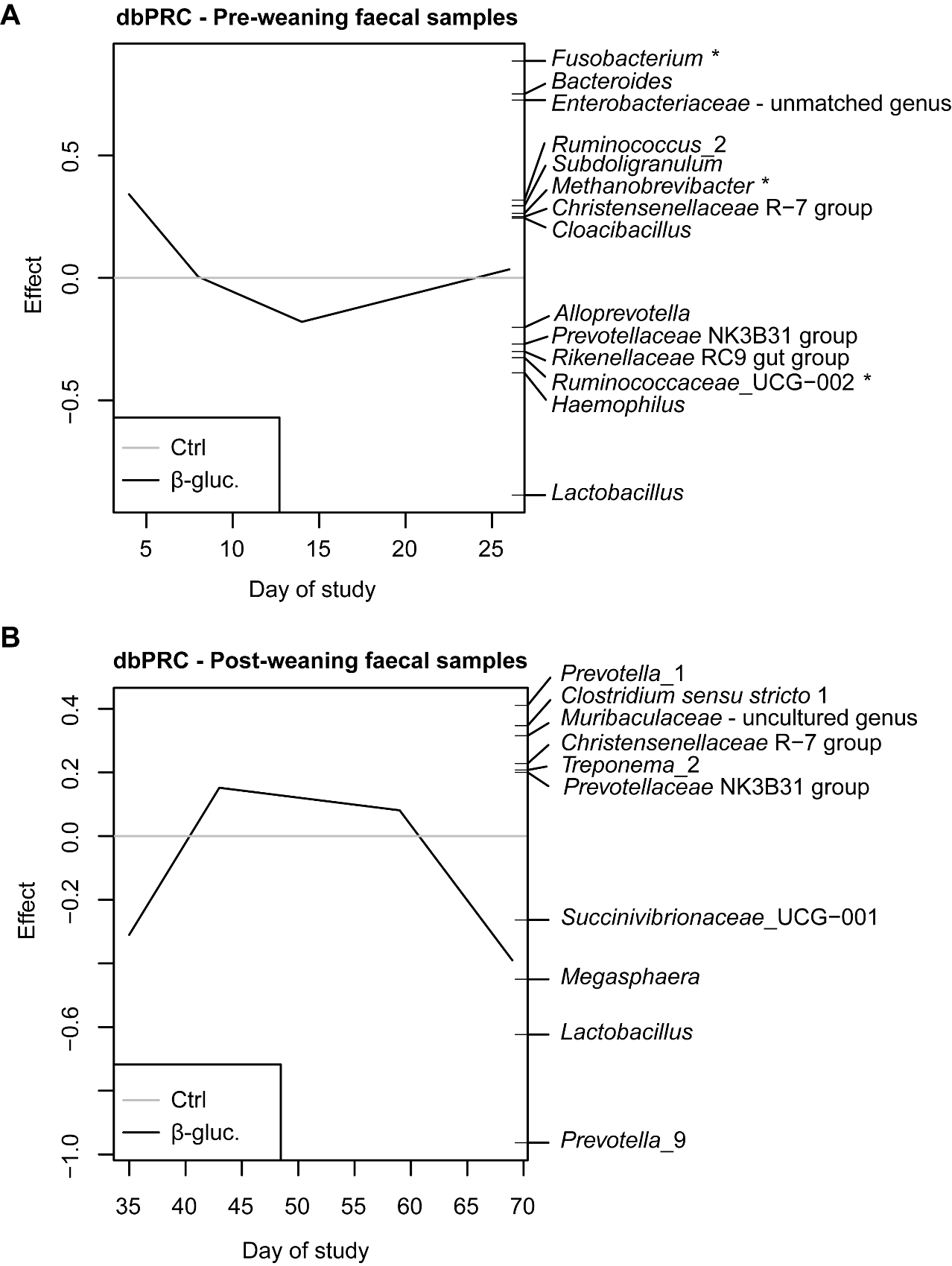


**Figure S4.** Weighted Unifrac distance-based Principal Response curves (dbPRC) of differences in pre-weaning (**A**) and post-weaning (**B**) faecal microbiota composition between β-glucan (black line) and control (reference baseline with zero PRC values) animals. The horizontal axis represents time, and the vertical axis represent PRC score values. Calculations were performed at the genus level, genera with a score lower than -0.2 or higher than 0.2 are shown on the right y-axis. Asterisks represent genera that were significantly differentially abundant.


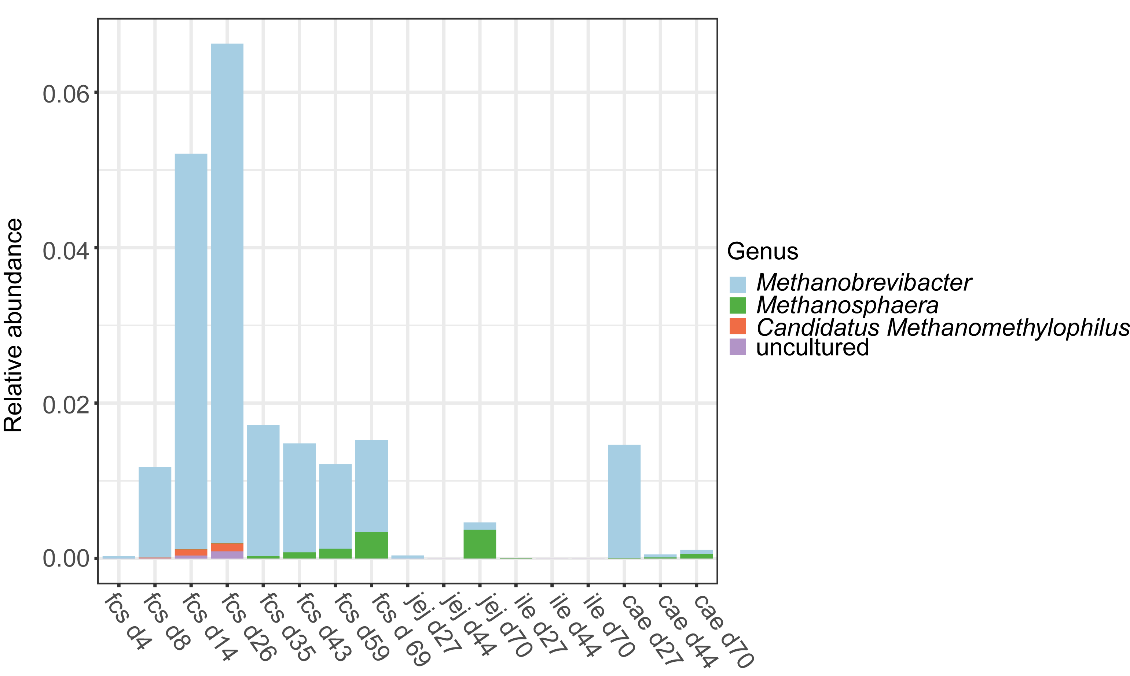


**Figure S5.** Taxonomic composition of *Archaea* within the piglet faeces and digesta over time. Data are given as the mean relative abundance at the genus level by sampling time point (d4-70) and by faeces (fcs) or gut segment; jejunum (jej), ileum (ile), caecum (cae). Data includes samples from both treatment groups.


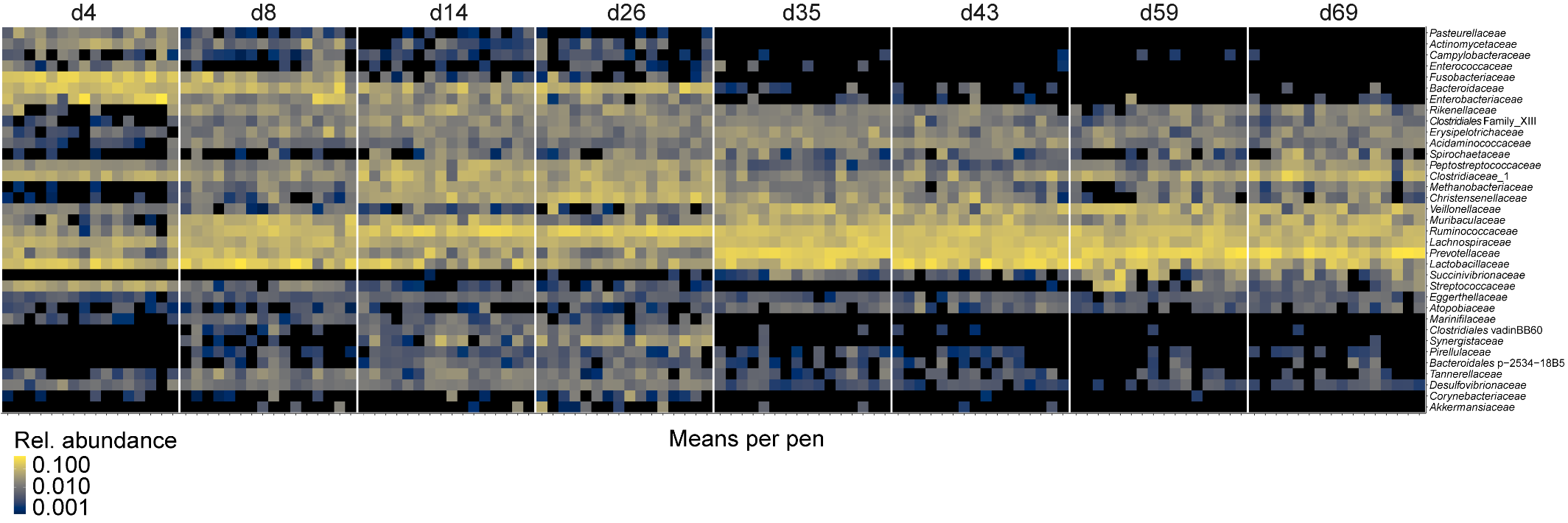


**Figure S6.** Heatmap of the relative abundance of the 35 most prevalent families in faecal samples over time. Each time point includes the means of 16 pens (8 control pens and 8 β-glucan pens).


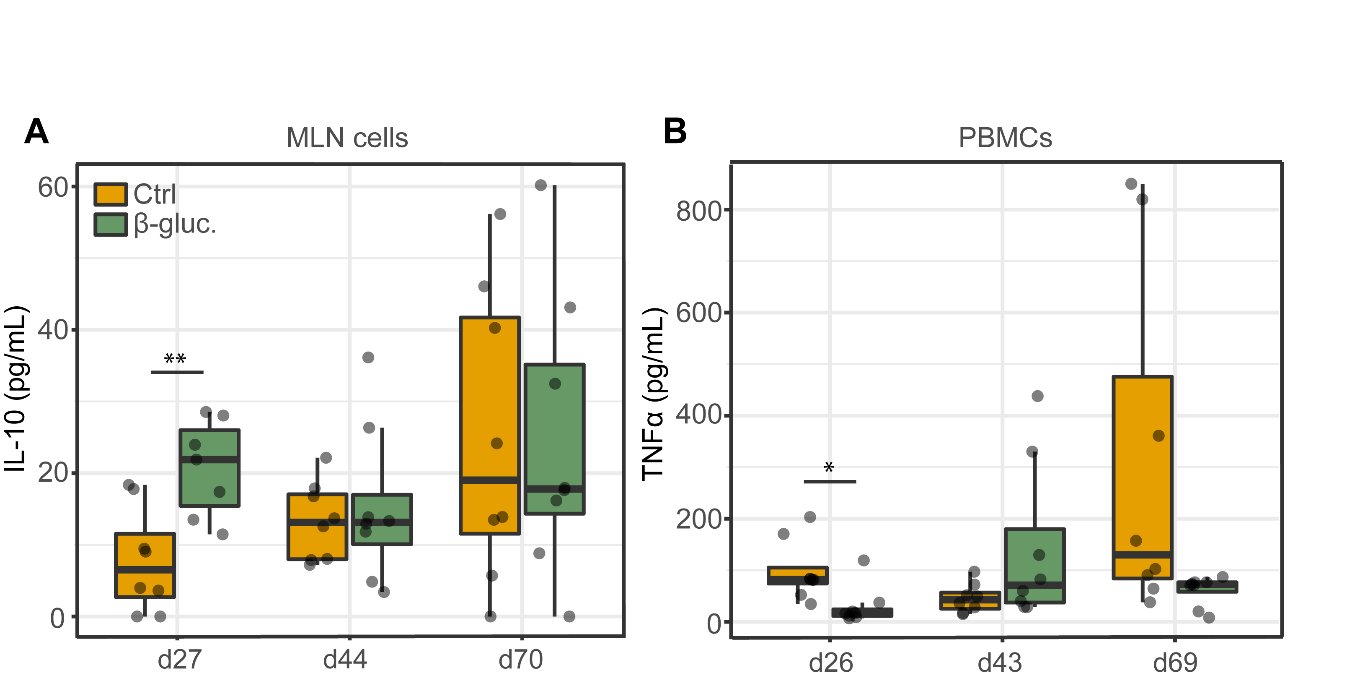


**Figure S7.** Levels of cytokines IL-10 (**A**) and TNFα (**B**) from LPS stimulated MLN cells and Con-A stimulated PBMCs, respectively. MLN cells and PBMCs were stimulated with 1 µg/mL of LPS or 2.5 µg/mL of Con-A for 24 h. Significant differences between treatments are indicated by asterisks (**; P<0.01 and *; P<0.05). Every dot represents a single animal (n = 7 or 8 per group) and error bars represent standard deviations. Statistical analysis was performed for every time point (T-test) and over time (Two-way ANOVA). Data were checked for normal distribution and equal variances and log-transformed when required.


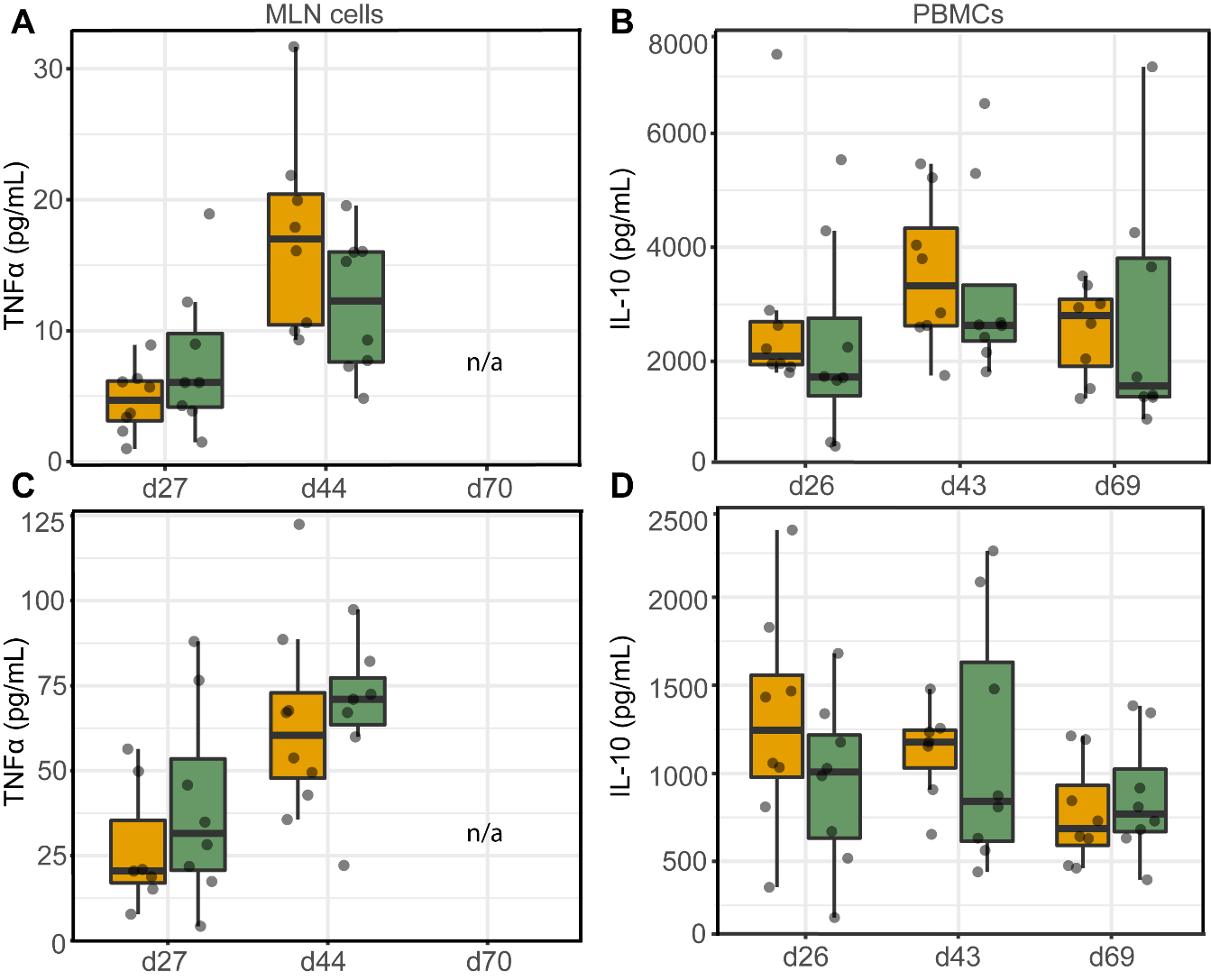


**Figure S8.** Levels of cytokines TNFα (**A**, **C**) and IL-10 (**B**, **D**) from stimulated MLN cells and PBMCs. MLN cells and PBMCs were stimulated with 10 µg/mL LPS (**A**, **B**) or 5 µg/mL Con-A (**C**, **D**) for 24 h. Every dot represents a single animal (n = 7 or 8 per group) and error bars represent standard deviations. Statistical analysis was performed for every time point (T-test) and over time (Two-way ANOVA). Data were checked for normal distribution and equal variances and log-transformed when required. No cytokine levels were detected on day 70 (MLN cells) as indicated by n/a.


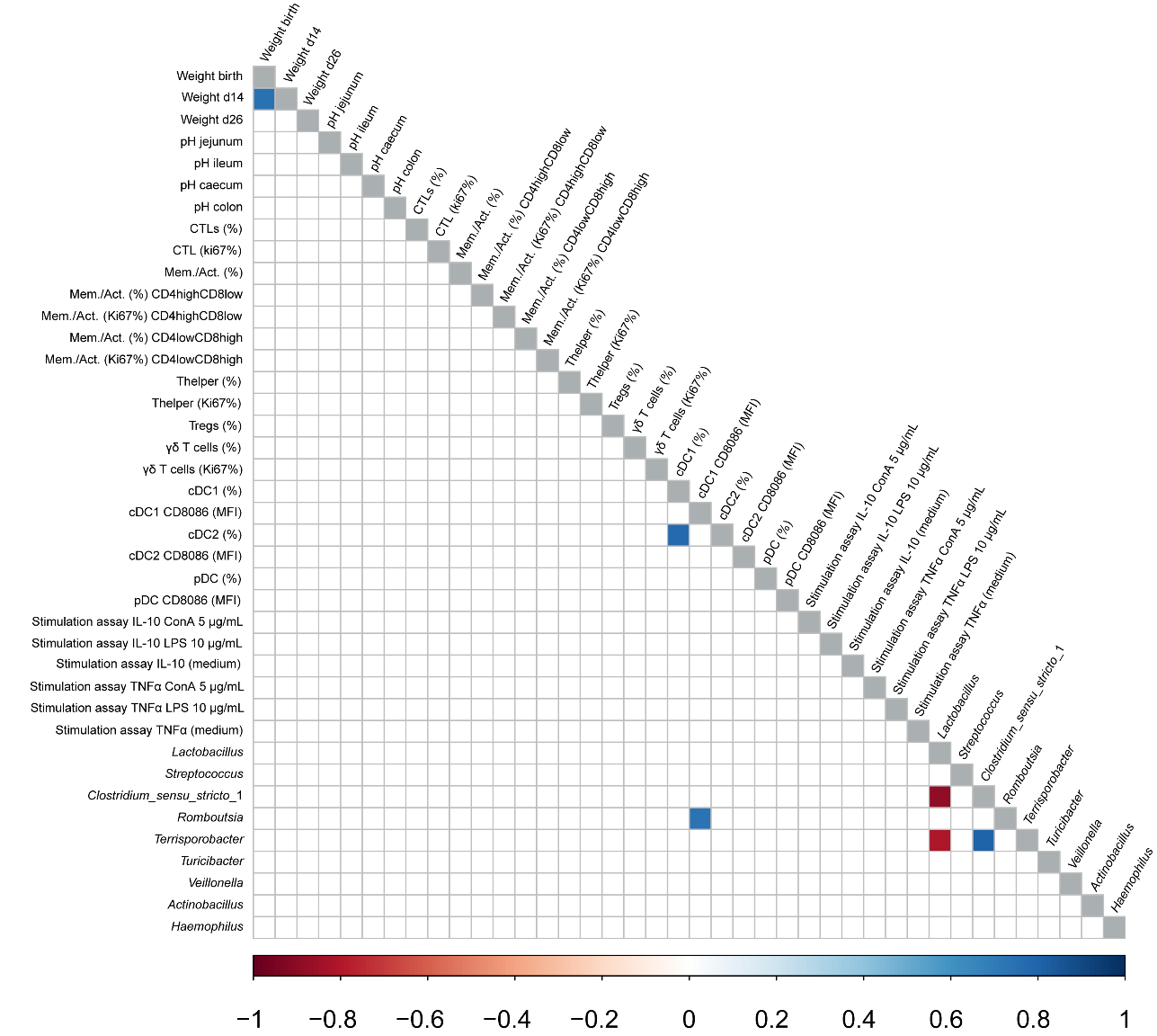


**Figure S9.** Correlation plot of several study parameters, including physiological parameters (weight and pH), immunological parameters (MLN cell analysis and stimulation assay) and ileal microbiota composition abundances (genus level). This figure only includes data from day 27 of the study. All correlations with an adjusted p-value below 0.05 are shown in the correlation plot. Color intensity is proportional to the correlation coefficient.
