# Supplementary material for: Impact of Yeast-Derived β-Glucans on the Porcine Gut Microbiota and Immune System in Early Life
Source: Microorganisms. 2020 Oct 13;8(10):1573. doi: 10.3390/microorganisms8101573 (PMC7601942; doi:10.3390/microorganisms8101573)
Supplement: Supplementary file 1 [file microorganisms-08-01573-s001.zip › Table S1.docx]

**Table S1.** Experimental diets fed during the experimental period.

|  | **Weaner Diet**  Day 25-44 | **Nursery Diet**  Day 45-70 |
| --- | --- | --- |
| *Ingredient composition (%)* |  |  |
| Barley | 25.00 | 30.00 |
| Wheat | 25.00 | 22.00 |
| Corn | 15.28 | 12.00 |
| Soybean meal (48% crude protein) | 10.00 | 9.90 |
| Sweet whey powder | 9.29 | 4.29 |
| Soy protein concentrate ^1^ | 4.30 | 4.00 |
| Soya oil | 3.20 | 3.31 |
| Cane molasses (> 47.5% sugar) | 1.00 | 1.00 |
| Sucrose | 1.50 | 1.50 |
| Wheat bran | 1.50 | 5.13 |
| Sunflower seed meal (27% crude protein) | 0 | 3.00 |
| Sodium chloride | 0.58 | 0.46 |
| Sodium bicarbonate | 0 | 0.29 |
| Mono-calcium phosphate | 0.29 | 0.03 |
| Limestone (calcium carbonate) | 0.47 | 0.56 |
| Organic acids ^2^ | 0.15 | 0.15 |
| Phytase ^3^ | 0.05 | 0.05 |
| Vitamins and trace minerals ^4^ | 1.05 | 1.12 |
| Synthetic amino acids | 1.34 | 1.21 |
| Total | 100.00 | 100.00 |
|  |  |  |
| *Calculated nutrients, g/kg* |  |  |
| Moisture | 110 | 113 |
| Crude protein | 170 | 175 |
| Crude fat | 50 | 52 |
| Crude fibre | 28 | 40 |
| Crude ash | 47 | 47 |
| Starch (Ewers method) | 384 | 378 |
| Total dietary fibre | 141 | 165 |
| Soluble dietary fibre | 64 | 79 |
| Insoluble dietary fibre | 80 | 94 |
| Lactose | 65 | 30 |
| Calcium | 5.50 | 5.50 |
| Phosphorus | 4.50 | 4.36 |
| Digestible Phosphorus | 4.10 | 3.50 |
| Na | 3.00 | 3.00 |
| Cu (total, mg) | 165 | 167 |
| Zn (total, mg) | 124 | 129 |
| Metabolic energy (MJ) | 14.22 | 13.84 |
| Net energy (MJ) | 10.46 | 10.11 |
| Standardized ileal digestible lysine | 12.35 | 12.03 |
| *Analysed nutrients, g/kg* |  |  |
| Moisture | 101 | 97 |
| Crude protein | 170 | 176 |
| Crude fibre | 28 | 40 |
| Crude fat | 53 | 53 |
| Crude ash | 46 | 47 |
| Zinc (mg/kg) | 118 | 116 |

^1^HP 300 (Hamlet protein, Horsens, Denmark); ^2^ Fylax Forte HC-SP (Trouw Nutrition Selko, Tilburg, The Netherlands) ^3^ Phyzyme XP 5000 TPT (Danisco Animal Nutrition, Marlbourough, UK ) providing 600 FTU 6-phytase per kg feed; ^4^ Farmix (Trouw Nutrition, Putten, The Netherlands), provided per kg feed: 8000 IU vit A, 2000 IU vit D3, 100 (weaner) or 150 (nursery) IU vit E-acetate, 1.5 mg menadione, 1 mg thiamine mononitrate, 4 mg riboflavin, 1 mg pyridoxine, 30 µg cyanocobalamin, 20 mg niacin, 12 mg pantothenic acid, 300 µg folic acid, 150 mg choline chloride, 50 mg betain.
