# Supplementary figures and images for: Impact of Yeast-Derived β-Glucans on the Porcine Gut Microbiota and Immune System in Early Life
Source: Microorganisms. 2020 Oct 13;8(10):1573. doi: 10.3390/microorganisms8101573 (PMC7601942; doi:10.3390/microorganisms8101573)

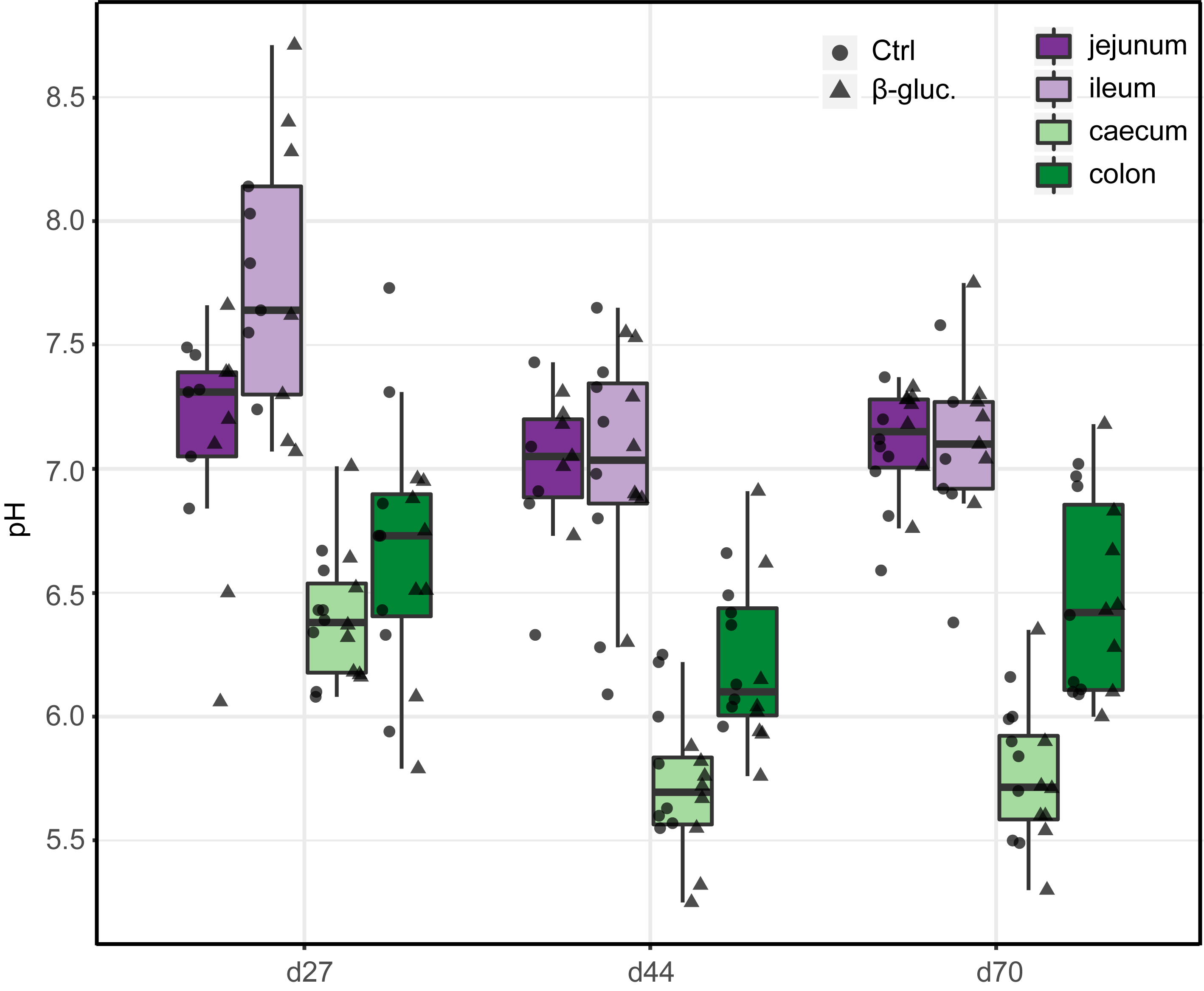

Supplement: Supplementary file 1 [file microorganisms-08-01573-s001.zip › Figure S1.tif]

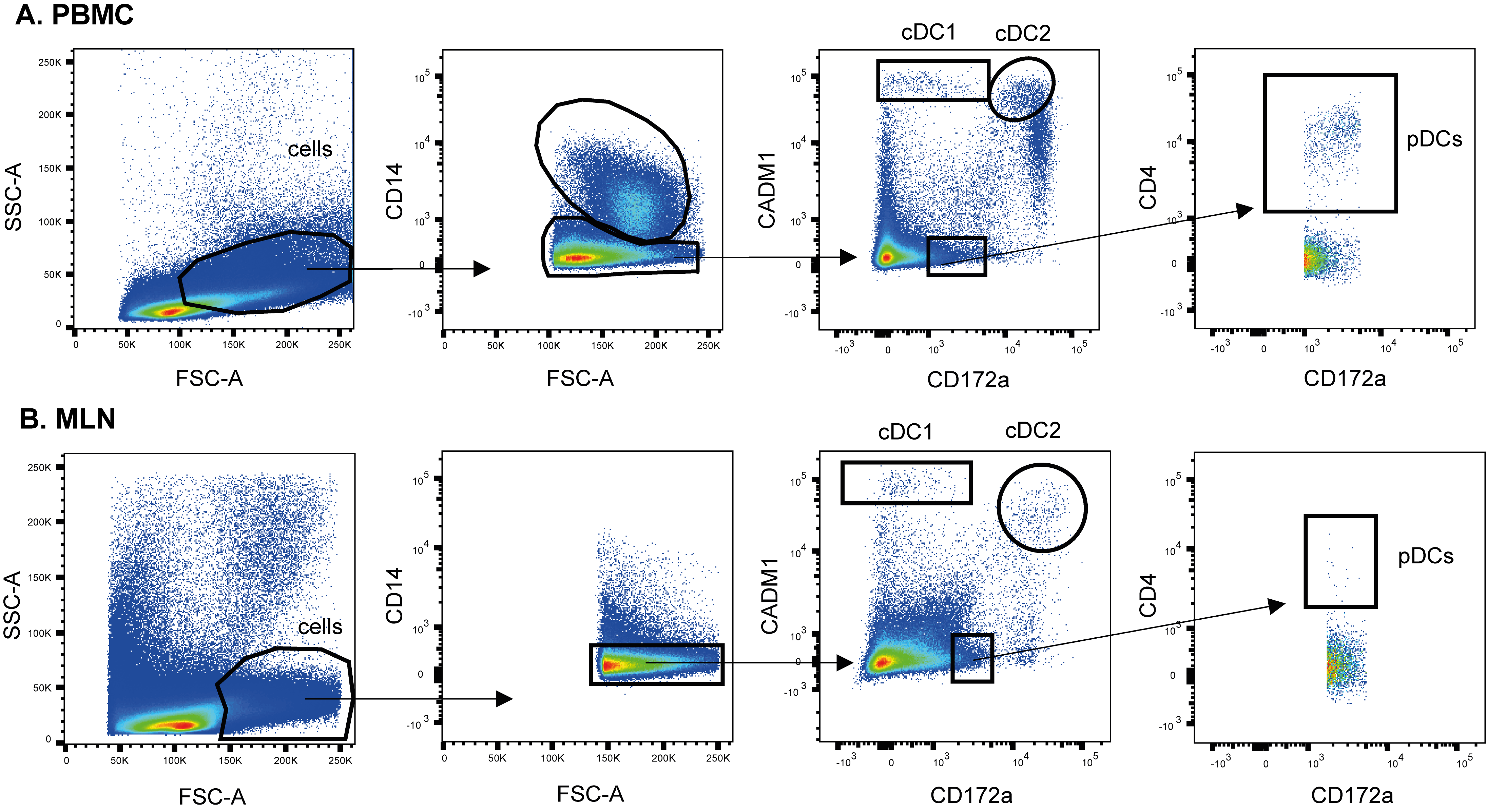

Supplement: Supplementary file 1 [file microorganisms-08-01573-s001.zip › Figure S2.tif]

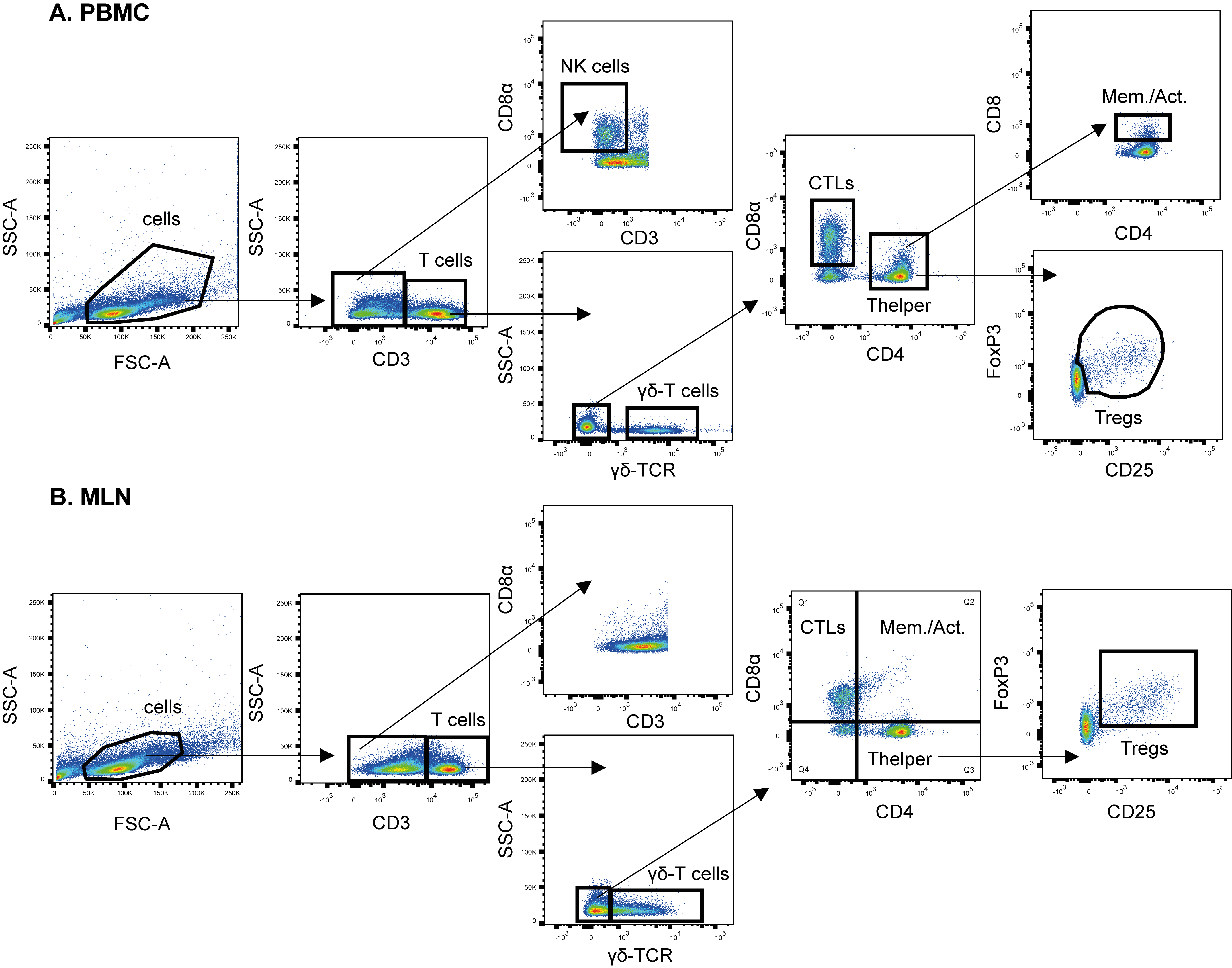

Supplement: Supplementary file 1 [file microorganisms-08-01573-s001.zip › Figure S3.tif]

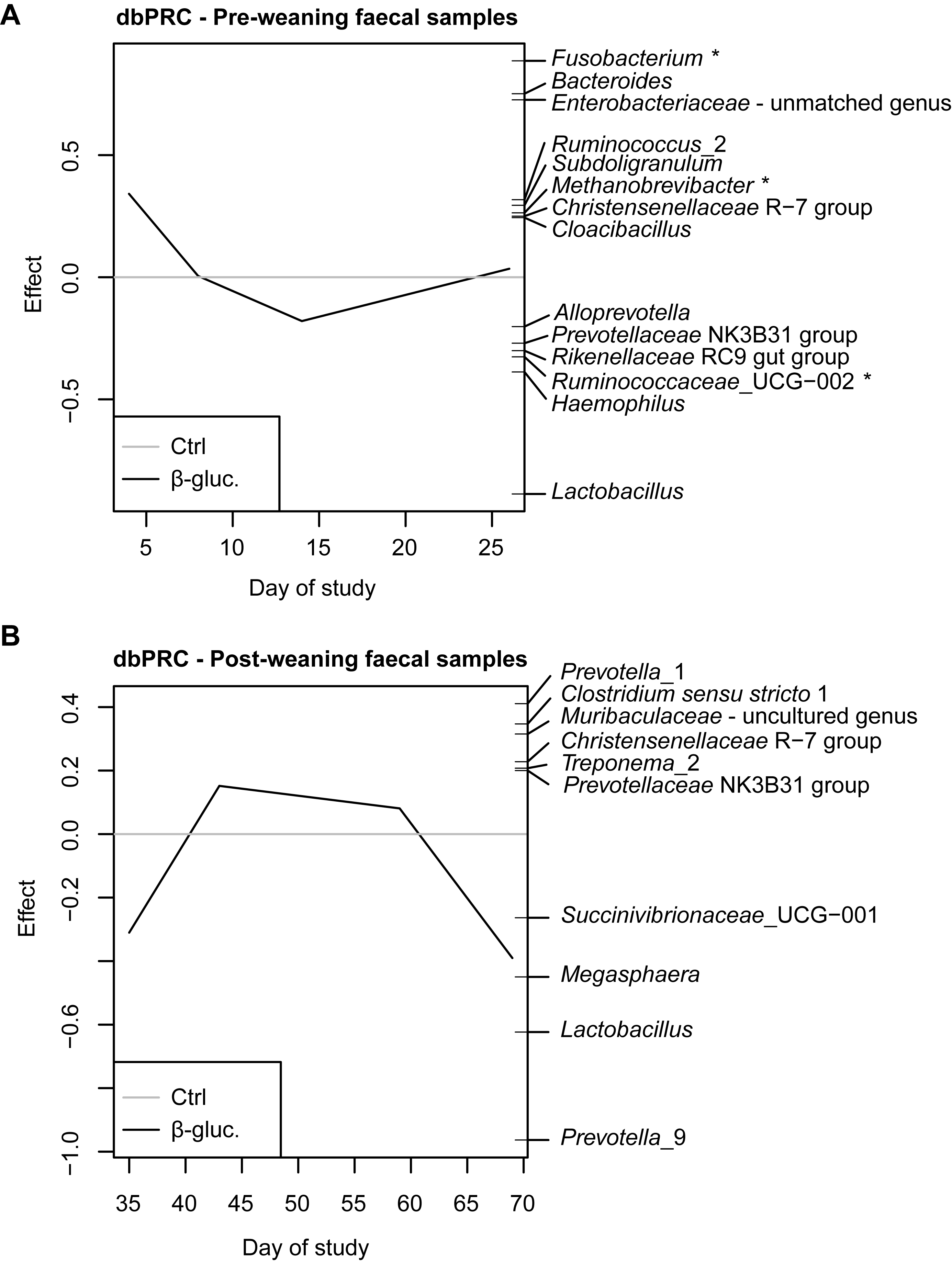

Supplement: Supplementary file 1 [file microorganisms-08-01573-s001.zip › Figure S4.tif]

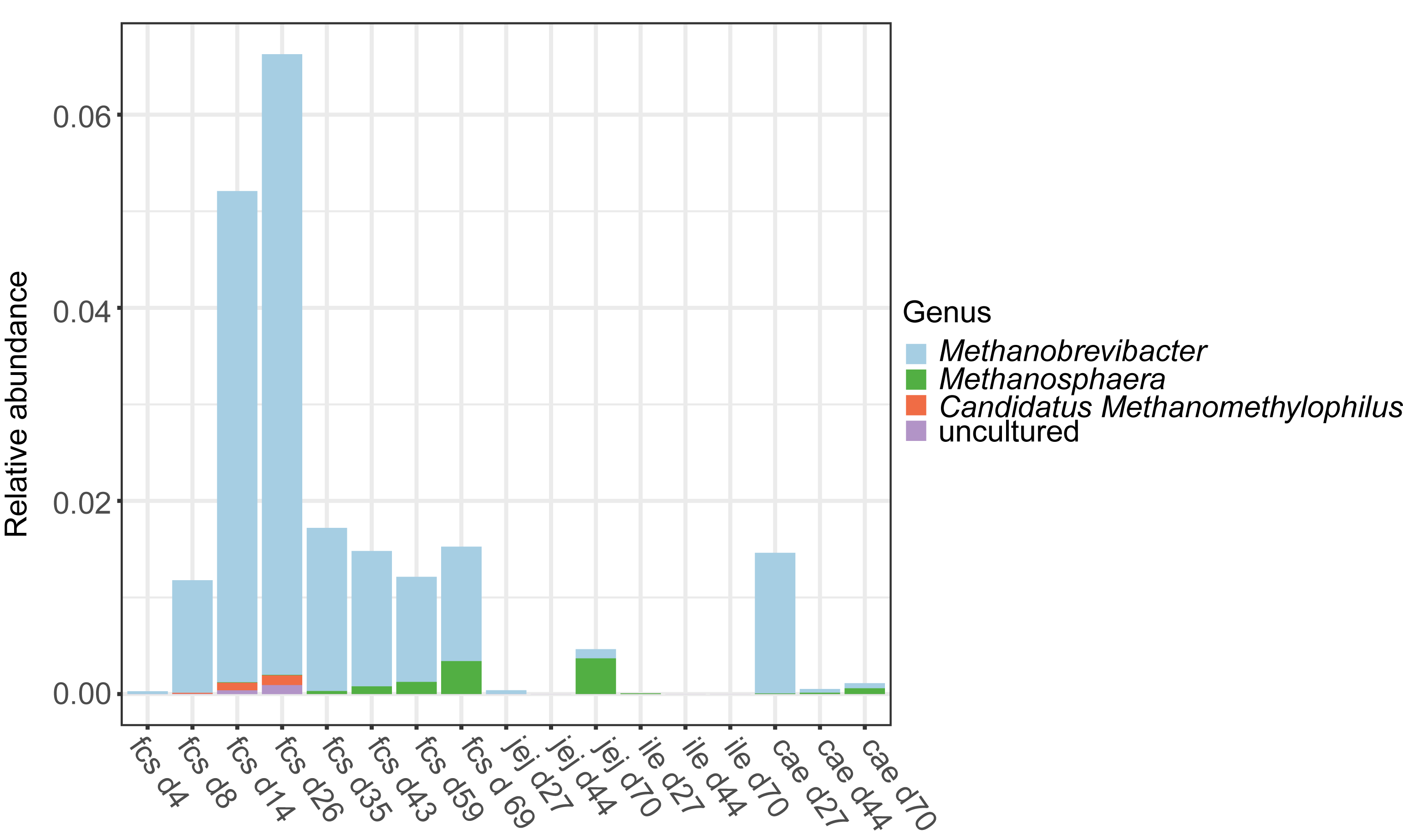

Supplement: Supplementary file 1 [file microorganisms-08-01573-s001.zip › Figure S5.tif]

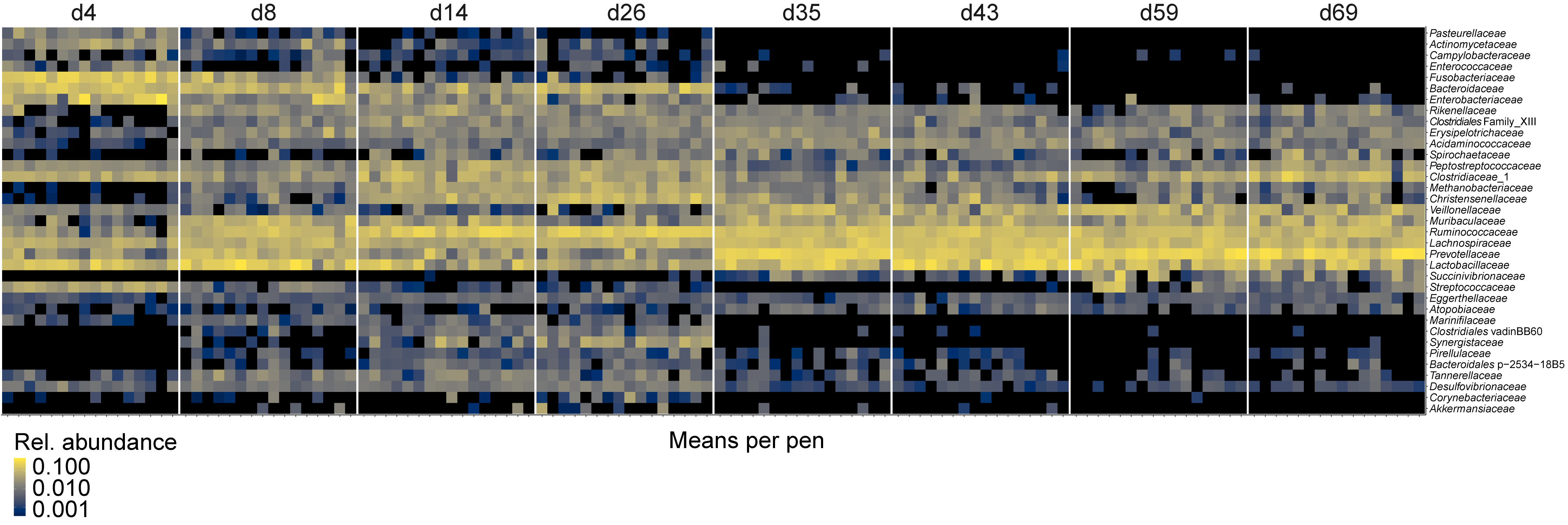

Supplement: Supplementary file 1 [file microorganisms-08-01573-s001.zip › Figure S6.tif]

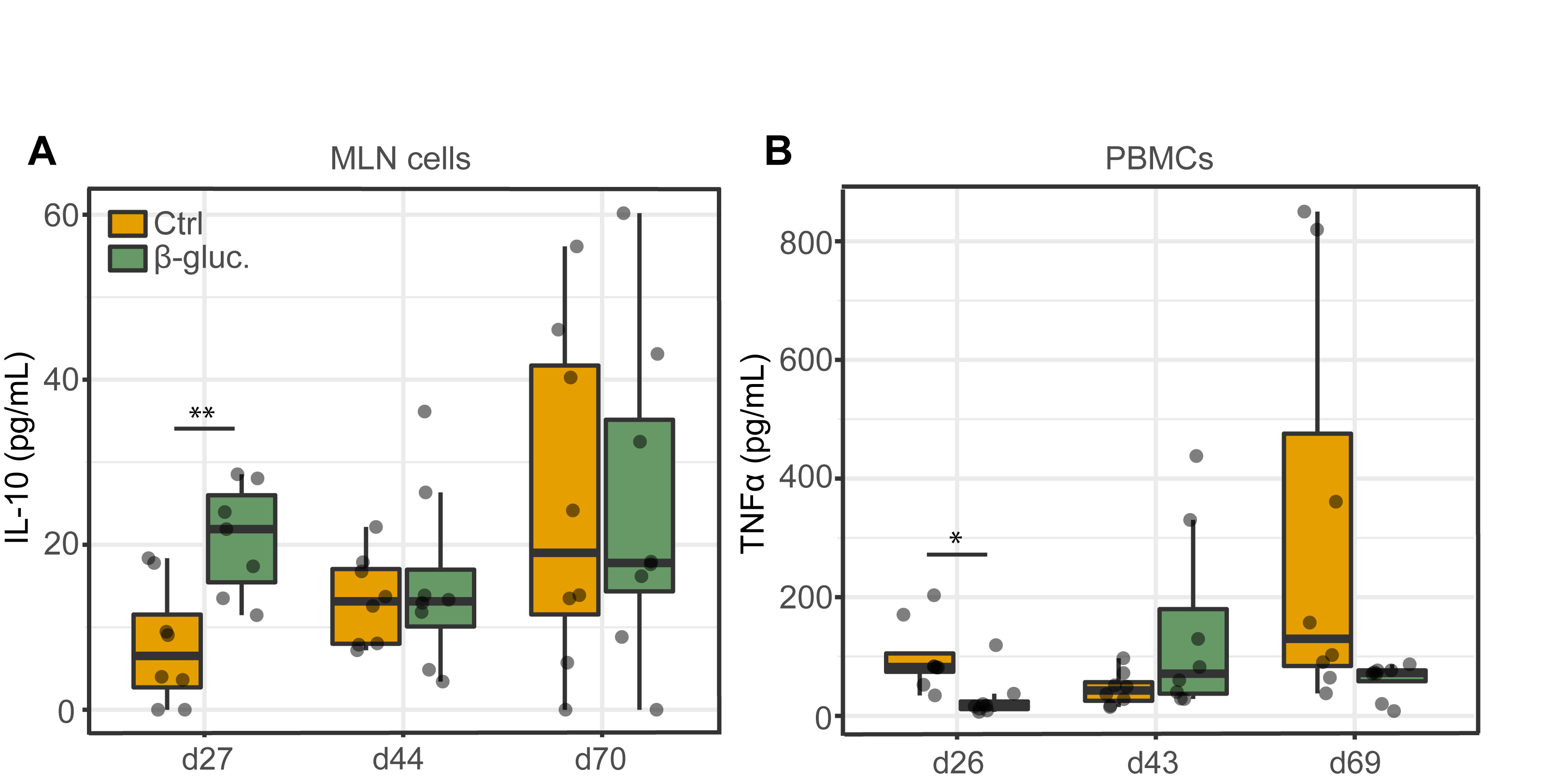

Supplement: Supplementary file 1 [file microorganisms-08-01573-s001.zip › Figure S7.tif]

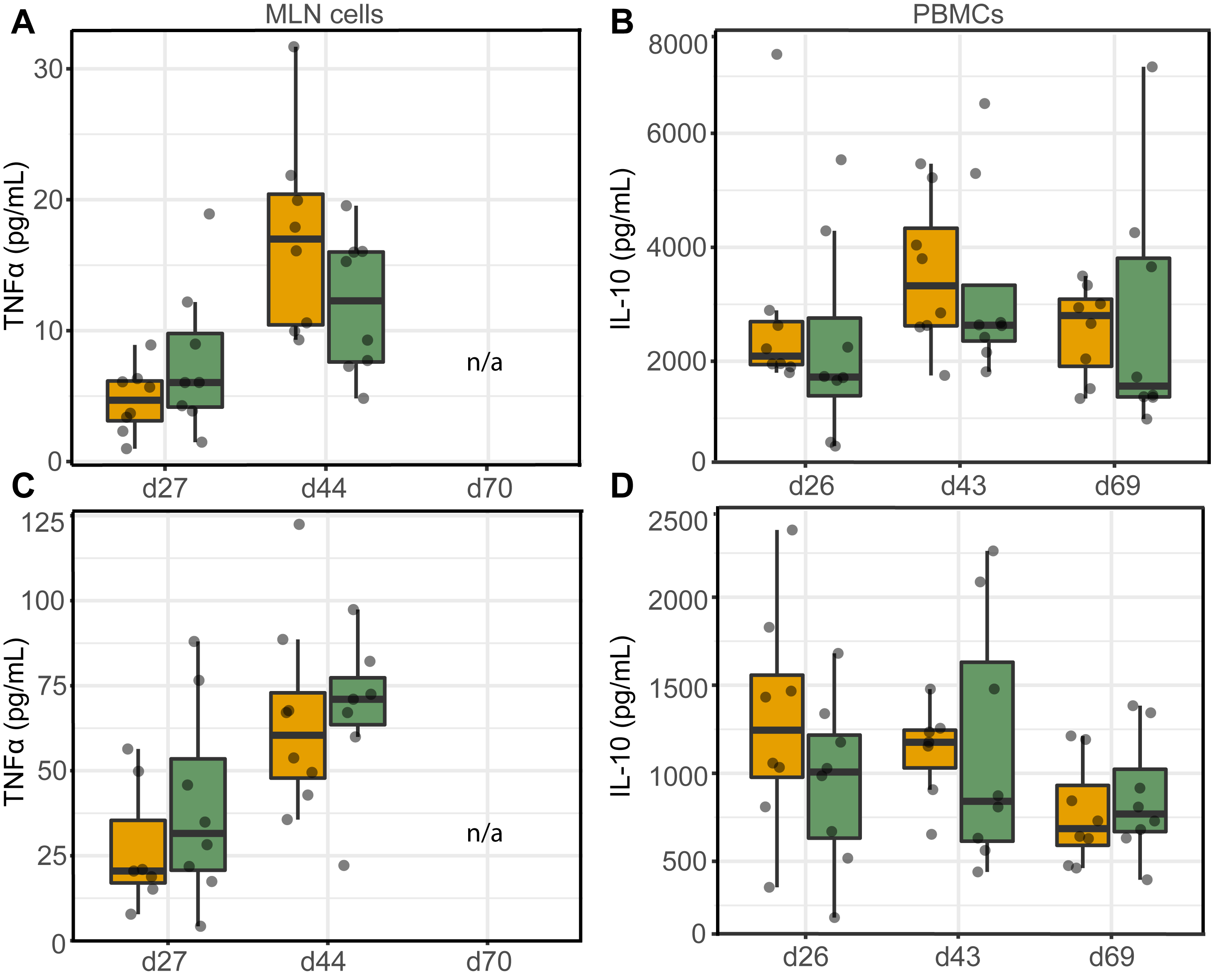

Supplement: Supplementary file 1 [file microorganisms-08-01573-s001.zip › Figure S8.tif]

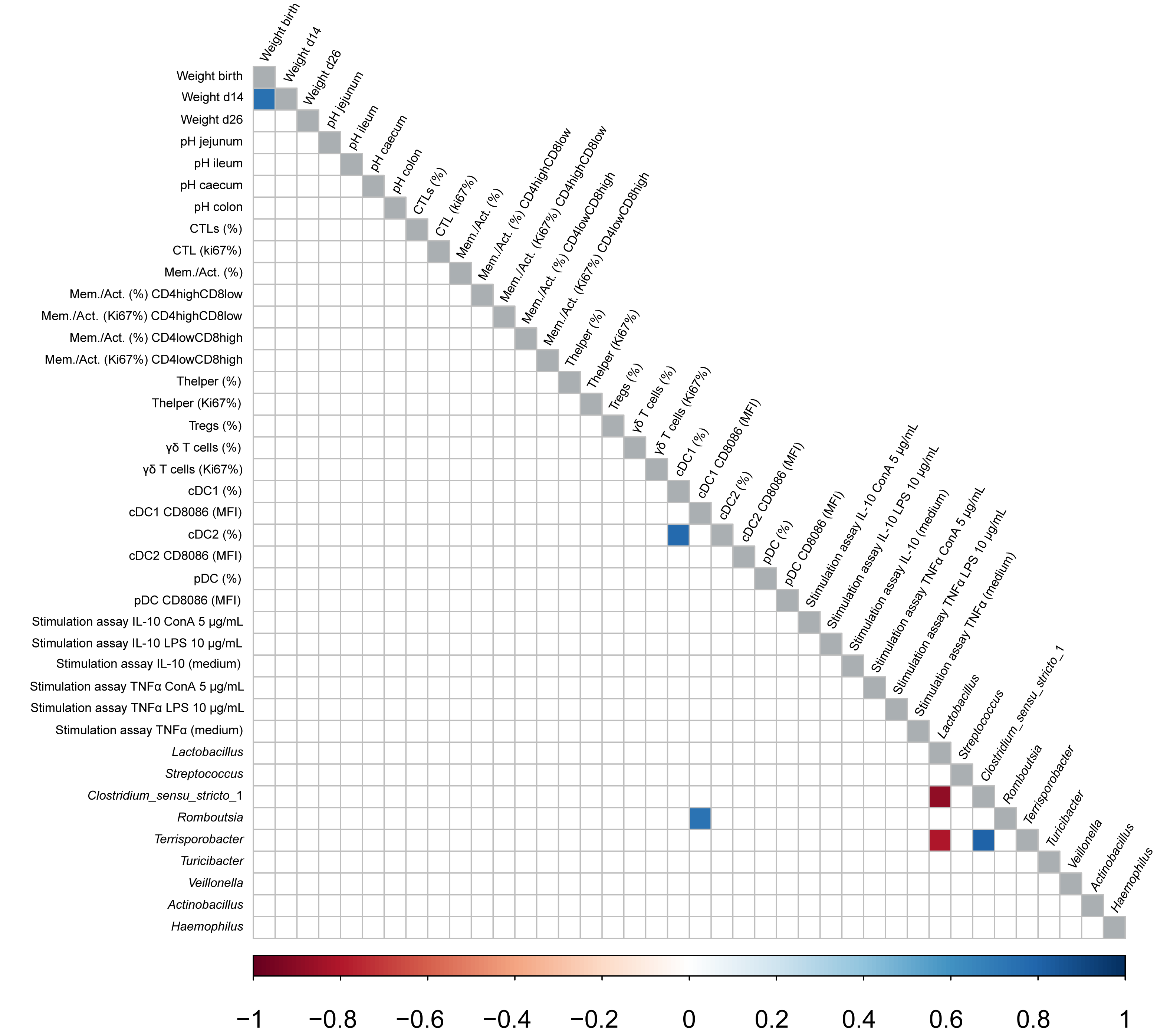

Supplement: Supplementary file 1 [file microorganisms-08-01573-s001.zip › Figure S9.tif]
